# Additives in Supplements for Grazing Beef Cattle

**DOI:** 10.3390/ani14243688

**Published:** 2024-12-20

**Authors:** Karine R. S. Naves, Kamila. A. K. Moraes, Lorrayne O. da Cunha, Natasha B. Petrenko, Juliana C. Ortelam, Jarliane N. Sousa, Caroline F. Covatti, Dener Nunes, Carla S. Chaves, Flávio L. de Menezes, André S. de Oliveira, Eduardo H. B. K. Moraes

**Affiliations:** 1 Núcleo de Estudos em Pecuária Intensiva—NEPI, Universidade Federal de Mato Grosso, Campus Universitário de Sinop, Sinop 78557-267, Brazil; karineribeiro_santos@hotmail.com (K.R.S.N.); kamilandreatta@gmail.com (K.A.K.M.); loorrayne.cunha@gmail.com (L.O.d.C.); julianacandeias@outlook.com (J.C.O.); jarlianezootecnista@gmail.com (J.N.S.); caroline_covatti@hotmail.com (C.F.C.); dener.nb@gmail.com (D.N.); carlazootecnia@gmail.com (C.S.C.); 2 Department of Animal Science, Colorado State University, Fort Collins, CO 80521, USA; natashabpetrenko@gmail.com; 3 Núcleo de Pesquisa em Melhoramento Animal—NUPEMA, Universidade Federal de Mato Grosso, Campus Universitário de Sinop, Sinop 78557-267, Brazil; flm.zotecnista@gmail.com; 4 Dairy Cattle Research Lab, Universidade Federal de Mato Grosso, Campus Universitário de Sinop, Sinop 78557-267, Brazil; andre.oliveira@ufmt.br

**Keywords:** active yeast, digestibility, functional oil, marandu palisadegrass, monensin, pasture

## Abstract

Pasture-based beef cattle production systems aim to maximize the interaction between forage and supplements to increase activity. In addition, supplementation and the use of food additives help optimize production efficiency and improve the use of additional nutrients. The aim of this study was to evaluate the effects of the use of additives in protein–energy supplements (PESs) on the intake, digestibility, and ruminal parameters of beef cattle raised on pasture in the rearing phase. Five male cattle were allocated to a 5 × 5 Latin square design, with five experimental periods and five treatments. The animals were kept under continuous grazing in individual paddocks of marandu palisadegrass (*Urochloa brizantha* cv. Marandu). The treatments consisted of a mineral mixture (MM) (0.080 kg/animal/day), PES (4.0 kg/animal/day), PES with monensin sodium (120 mg/animal/day), PES with functional oil (2500 mg/animal/day) and PES with active yeast (4000 mg/animal/day). PESs increased the total dry matter intake (DMI) (kg/day) and the DMI in terms of % body weight (%BW) and increased the intake of crude protein (CP) and the dry matter intake of digestible organic matter (DOM). The use of additives did not affect nutrient intake or digestibility. Compared with MM, the PES increased the concentrations of ruminal ammonia nitrogen and serum urea nitrogen. In addition, there was an increase in fecal nitrogen, retained nitrogen, microbial nitrogen synthesis and microbial efficiency. The use of monensin sodium, functional oil and active yeast in protein–energy supplements for beef cattle in pastures did not affect the nutritional parameters under the conditions observed in this study.

## Simple Summary

Nutritional strategies such as supplementation may be effective in maintaining the productivity of beef cattle raised on pastures. To optimize animal production, feed additives are used together with supplements to ensure an interaction between basal and supplementary resources. This study aimed to evaluate how additives in protein–energy supplements (PESs) improve beef cattle raised on pasture. Five cattle were tested via a 5 × 5 Latin square design, with treatments including a mineral mixture (MM) and a PES with or without additives such as monensin sodium, functional oil, or active yeast. Compared with MM, the PES increased total dry matter intake (DMI), crude protein intake, and nitrogen levels. However, the additives did not significantly impact nutrient intake or digestibility. The PES also increased microbial nitrogen synthesis and efficiency.

## 1. Introduction

Supplementation with concentrate for beef cattle raised on pasture is used to improve the efficiency of nutrient use by the ruminal microbiota and has been frequently adopted by nutritionists. Supplementation becomes even more necessary in the autumn and winter periods in Central Brazil, which experiences long periods of water deficit, with a consequent reduction in the quality and quantity of available forage. Combined with the use of supplementation, feed additives are important dietary tools for increasing the ruminal efficiency, productivity and profitability in grazing systems [1,2,3].

Ionophores (antibiotics), such as sodium monensin, are known to promote changes in the ruminal microbiota, such as increased propionate production, maintenance of fermentation and ruminal pH, improved feeding efficiency in ruminants and reduced methane emission [4,5,6].

According to Argudín et al. [7], the inappropriate use of antibiotics in food animals has contributed to the emergence of bacteria resistant to their effects. Despite the positive results obtained in animal production with the use of ionophores such as monensin, there is a public health concern regarding the use of antibiotics in animal feed due to possible antibiotic resistance in human pathogenic bacteria. As a result, in 2006 the European Union banned the use of antibiotics as growth promoters in animal feed. This restriction resulted in a global effort to develop alternative additives to monensin and antibiotics in general. In this scenario, the search for alternatives to monensin gains relevance, especially due to its ban in the European Union, motivated by concerns related to public health and leading to the investigation of natural products that may have antibiotic-like effects [8]. Important alternatives to antibiotics reported in livestock include enzymes, organic acids, probiotics, prebiotics, yeasts and functional oils [3,9,10].

Several studies have shown that functional oils are plant extracts that manipulate ruminal fermentation, mitigate methane emissions and increase the proportion of ruminal propionate [9,11,12]. Furthermore, functional oils alter the profile of volatile fatty acids in the rumen, resulting in a reduction in the acetate:propionate ratio but not in total production [12,13].

Active yeast is a probiotic additive, and for ruminants there is a reduction in ammonia concentration and an increase in the number of microorganisms, which can lead to an increase in microbial synthesis and efficiency and a greater flow of amino acids to the intestine [14,15]. Yeasts increase the initial rate of dry matter (DM) digestion, stabilize the ruminal pH, reduce the redox potential of the rumen and change the microbial population [16,17,18].

However, the random addition of additives as supplementary resources without considering specific situations or targeting the substrate may result in unsatisfactory results. Although much progress has been made in advancing additive technology for beef cattle, studies are still needed to reduce response variability, especially under grazing conditions, where knowledge of the effects of additives on nutritional parameters is still incipient.

In this context, we propose the hypothesis that the use of additives in protein–energy supplements improves the nutritional parameters of beef cattle during intensive rearing on pasture. Therefore, our objective was to evaluate the effects of protein–energy supplement additives on the intake, digestibility and ruminal parameters of beef cattle in the rearing phase.

## 2. Materials and Methods

The procedures involving animals were approved by the Committee on Ethics in the Use of Animals (CEUA) of the Universidade Federal de Mato Grosso (Process N°. 23108.920449/2018-21).

The experiment was conducted at the Paraíso Silvestre farm in the municipality of Sinop, Mato Grosso, Brazil (11°55′17″ S, 55°27′29″ W), from 4 June to 17 September 2020. Chemical analyses were carried out at the Laboratório do Núcleo de Estudos em Pecuária Intensiva (NEPI) at the Universidade Federal do Mato Grosso (UFMT), Sinop University Campus.

### 2.1. Experimental Design, Animals and Supplements

Five noncastrated male Nelore cattle with an initial average body weight of 355 ± 30 kg was used; the cattle were distributed in a 5 × 5 Latin square design with five experimental periods and five treatments. The animals were kept under continuous grazing in individual paddocks of 0.5 hectares (ha) formed with the grass *Urochloa brizantha* cv. Marandu and equipped with individual drinking fountains and feeders. The animals were weighed and randomly distributed in the paddocks at the beginning of each experimental period. To minimize the possible effects of the paddocks on the treatments, the animals were rotated between the five paddocks during each experimental period.

The treatments consisted of a mineral mixture (MM–control treatment) (0.080 kg/animal/day), a protein–energy supplement (PES) (4.0 kg/animal/day) (Table 1), PES with sodium monensin Rumensin™ (Elanco, São Paulo, Brazil) at a dose of 120 mg/animal/day, PES with Essential^®^ functional oil (Oligo Basics Agroind. Ltda., Cascavel, Brazil) at a dosage of 2500 mg/animal/day and PES with the active Yea-Sacc (Alltech, Maringá, Brazil) at a dosage of 4000 mg/animal/day.

The supplements were formulated to be isonitrogenated, containing 200 g/kg crude protein (CP), and were offered daily at 10 am. The additives were added directly to the animals through the topdress and the doses used were based on the manufacturers’ recommendations.

### 2.2. Experimental Procedures and Sampling

Each experimental period lasted 21 days, with 15 days for adaptation to the supplement and six days for sample collection. On the first day of each experimental period, pasture samples were collected to determine the chemical composition and availability of total DM. Forage mass was measured by cutting, with pruning shears, four samples per paddock with the aid of a metallic quadrangular frame (0.5 m × 0.5 m, 1 m^2^ paddock/period) at a residual height of 5 cm from the ground.

The samples were weighed and dried in a forced ventilation oven (55 °C) for 72 h, after which dry matter (DM) analysis was carried out in a 105 °C oven. The dry weight of each sample was multiplied by the paddock area to estimate the total forage mass. The paddocks had an average forage availability of 3383.33 ± 967 kg DM/ha. To evaluate the chemical composition of the pasture consumed by the animals, samples collected via manual grazing simulation were collected from all paddocks on the 16th day of each experimental period. The samples were dried in a forced ventilation oven (55 °C) for 72 h and processed in a “Willey” knife mill.

Fecal excretion was estimated on the basis of the concentration of titanium dioxide (*TiO*_2_) as an external indicator:

(1) $$FE=TiO_{2}\;provided\frac{TiO_{2}\;provided\left(g\right)}{TiO_{2}fecal\left(g{kg}^{-1}DM\;fecal\right)}\;$$

*TiO*_2_ (15 g/day) was applied via an esophageal tube at 9 am from the 11th to the 18th day of each experimental period. Four stool samples were collected on the 16th day at 4 pm, on the 17th day at 2 pm, on the 18th day at noon and on the 19th day at 8 am; the samples were dried in a forced ventilation oven (55 °C) for 72 h, after which a composite sample was formed for each animal per experimental period.

On the 20th day at 2 pm for each experimental period, spot urine samples were collected. Aliquots of 10 mL were diluted in 40 mL of 0.036 N sulfuric acid (H_2_SO_4_) and frozen (−20 °C) for subsequent quantification of creatinine, urea and purine derivative (PD) contents. The concentrated urine aliquots were frozen (−20 °C) to determine total nitrogen. On the 20th experimental day, blood was also collected via coccygeal puncture with test tubes containing separating gel and a coagulation accelerator (Greiner Bio-One VACUET^®^, Americana, SP, Brazil). The samples were centrifuged (3000 rpm for 15 min) to obtain serum, which was frozen (−20 °C) to determine the serum urea nitrogen (SUN) concentration.

At 2 pm on the 21st day, samples of rumen fluid were collected with the aid of a rumen probe and the samples were filtered through layers of gauze. The pH and temperature of the rumen fluid were measured immediately after collecting via a digital tool meter. Aliquots of 50 mL were mixed with 1 mL of sulfuric acid (H_2_SO_4_) (1:1) and frozen (−20 °C) for subsequent quantification of the ruminal ammonia nitrogen (RAN) concentration.

### 2.3. Laboratory Analyses

Samples of ingredients, pasture and feces were predried in a forced ventilation oven (55 °C) and subsequently processed in a “Willey” knife mill with 1 and 2 mm sieves for chemical analysis. The samples were analyzed according to the analytical procedures of the National Institute of Animal Science and Technology [19] for the following chemical compositions: DM, mineral matter (MM), CP (obtained by determining total nitrogen via the Kjeldahl method), neutral detergent insoluble fiber (NDF), neutral detergent insoluble protein (NDIP) and neutral detergent insoluble ash (NDIA).

To analyze the NDF concentration, the samples were treated with thermostable alpha-amylase without the use of sodium sulfite, which was corrected for ash residue [20] and nitrogenous compound residue (NDFap) [21].

Samples of the ingredients, pasture and feces processed at 2 mm were used to evaluate the indigestible neutral detergent fiber content (iNDF) [22]. iNDF was quantified via an in situ incubation procedure with nonwoven fabric (NWF) bags for 288 h. After the incubation period, the bags were washed and NDF analysis was continued [19].

To determine fecal production, the *TiO*_2_ concentration in the fecal samples was analyzed via the colorimetric technique [19] and the concentration in the samples was related to the daily dose of the indicator.

The *DMI* was estimated by quantifying the relationship between total fecal excretion and the indigestible fraction; therefore, the NDFi was used as an internal indicator, as follows:

(2) $$DMI=\frac{\left[\left(FE\;\times \;CIF\right)-IS\right]}{CIFO}+CDMS\;$$

where *FE* is the fecal excretion (g DM/day), *CIF* is the concentration of NDFi in the feces (g/g), *IS* is the amount of NDFi ingested in the supplement (kg/day), *CIFO* is the iNDF concentration in the forage (kg/kg) and *CDMS* is the supplemental DM intake (kg/day).

The apparent digestibility coefficient (*ADC*) was calculated via the following formula:

(3) $$ADC=\frac{\left[\left(\;ingested-excreted\right)\right]}{ingested}\times 100\;$$

The RAN concentration was determined via the indophenol-catalyzed colorimetric reaction method [23]. Blood serum was analyzed for urea content to obtain SUN via commercial kits (Gold Analisa Diagnóstica Ltd.a. Belo Horizonte, Brazil).

The creatinine, uric acid and urea contents of the urine samples were analyzed via commercial kits (Gold Analisa Diagnóstica Ltd., Belo Horizonte, Brazil). The urinary volume was estimated on the basis of the creatinine content according to previous methods [24]. The allantoin content was obtained from diluted urine samples according to the methodology of [25].

Total PD excretion (mmol/day) was obtained by summing the urinary excretion of allantoin and uric acid. Absorbed purines (APs, mmol/day) were calculated from PD excretion according to [26]

(4) $$AP=\frac{PD-0.301\;\times \;{BW}^{0.75}}{0.80}\;$$

where 0.301 × *BW*^0.75^ represents the excretion of endogenous *PD* and 0.80 represents the recovery of *AP* as *PD* in the urine.

The ruminal synthesis of nitrogen compounds (g *Nmic*/day) was calculated as follows:

(5) $$Nmic=\frac{70\;*\;AP}{0.93\;*\;0.137\;*\;1000}\;$$

where 70 is the nitrogen content in purines (mg/mmol), 0.93 is the true digestibility of purines and 0.137 is the average purine nitrogen:total nitrogen ratio in bacteria isolated in the rumen [26].

The microbial synthesis efficiency was calculated as the ratio of microbial protein to DOM (g of CPmic/kg of DOM).

Nitrogen excretion in urine was calculated by the concentration in spot samples and the urinary volume. Nitrogen retention was determined by the difference between ingested nitrogen, nitrogen excretion in feces and nitrogen excretion in urine.

### 2.4. Statistical Analyses

The data were analyzed via the GLM procedure of SAS, version 9.2 (Statistical Analysis System, version 9.2). The experimental design was a 5 × 5 Latin square with sources of variation in treatments, animals and experimental periods. The statistical model used was as follows:

(6) yijk=µ+Li+Cj+tk+€ijk 

where *yijk* = the observed value of the response variable; μ = overall average; *Li* = fixed effect related to the animal; *Cj* = fixed effect related to the period; *tk* = fixed effect relative to treatment; and *€ijk* = random experimental error. For all procedures, a significant level of 10% was adopted.

Comparisons between treatment methods were carried out according to the following orthogonal contrasts: (I) mineral mixture × protein–energy supplement (PES); (II) PES without additives × PES with additives; (III) PES with sodium monensin × PES with natural additives (functional oil and active yeast); and (IV) PES with functional oil × PES with active yeast.

## 3. Results

### 3.1. Dietary Intake

Compared with the mineral mixture, the PES increased the total DMI in kg/day (*p* < 0.01), total DM in %BW (*p* < 0.0001), total DM in CP (*p* < 0.0001), total DM in OM (*p* < 0.0001), total NDFap (*p* = 0.0981), and total DOM (*p* < 0.0001) (Table 2). Pasture DMI (*p* = 0.3139) and iNDF ( *p*= 0.5230) were not affected by the PES, with or without additives. However, there was no difference in the dietary intake of the additive sodium monensin, functional oil or active yeast compared with that of the PES.

### 3.2. Nutrient Digestibility

The PES increased the nutrient digestibility of DM (*p* = 0.0003), OM (*p* = 0.0003), CP (*p* < 0.0001), NDFap (*p* = 0.0322) and DOM (*p* < 0.0001) in relation to MM (Table 3). However, there was no difference in nutrient digestibility between the use of additives and the PES (Table 3).

### 3.3. Nitrogen Metabolism and Utilization

The PES increased the concentrations of RAN (*p* < 0.0001) and SUN in relation to those in the MM (*p* < 0.0001) (Table 4). Compared with the PES, the additives had no effect on RAN or SUN (*p* > 0.1).

The rumen pH decreased with the PES compared with MM (*p* = 0.0138). However, the PES with additives did not affect the ruminal pH (*p* > 0.1) (Table 4).

The PES increased ingested (*p* < 0.0001), fecal (*p* < 0.0001), urinary (*p* < 0.0001), and retained nitrogen (*p* < 0.0001) (Table 4) concerning MM. Fecal nitrogen (*p* = 0.082) was greater in the PES treatment group than in the PES with additives group (Table 4).

With PES, the ratios of N retained/N ingested (*p* < 0.0001) and N retained/N absorbed (*p* < 0.0001) were greater than those with MM (Table 4).

Compared with MM, the PES increased microbial nitrogen (*p* = 0.0001) and microbial efficiency (*p* = 0.0207) (Table 4). However, the PESs with additives did not affect nitrogen utilization efficiency (*p* > 0.1).

## 4. Discussion

Concentrated supplementation aims to complement the nutritional value of the available forage and becomes an ally for producers by increasing the efficiency of pasture use. However, depending on the level of supplementation, there are changes in the intake and digestibility of nutrients [27].

Supplementation may have contributed to the additive effects of animal intake, as the DMI (%BW) increased in the supplements, whereas the DMI in the pasture did not change.

The use of additives did not influence the voluntary intake of the animals in this study. These effects are similar to those reported by [28], who reported no change in total DMI or pasture DM with the use of sodium monensin in the supplementation of grazing heifers. The authors reported that the effectiveness of ionophore additives for beef cattle in pastures with low nutritional value is inconsistently presented in the literature.

Positive results can be achieved with the addition of monensin at relatively high concentrations by increasing the availability of substrate for rumen microorganisms. This effect was not observed in the present study since the total DMI did not differ between the PES with or without additives.

In contrast to what was observed in this study [29], a meta-analysis revealed an increase in DMI when monensin was replaced with functional oil in the diet of beef cattle. In other works [30,31], it was concluded that supplementation with functional oil provided greater DMI than monensin did.

The type of functional oil and the dose used are related to the effect on the DMI; low doses tend to stimulate intake and higher doses can negatively affect intake by influencing the palatability of the food [32]. Ref. [33] emphasized that the effects obtained are dependent on the type of oil and the dose used but stated that the type of diet associated with the functional oil and the animal’s growth phase also influence the response to the use of additives.

In a meta-analysis [34], it was reported that a dose of 10 g/day yeast reduced the DMI of cattle. On the other hand, the dose of 6 g/day resulted in a greater average daily gain. Thus, the authors concluded that doses above 6 g/day do not increase performance effects and may even depress these indicators. Although the dose of active yeast used in the present study was 4 g/day, it had no influence on the variables analyzed. Notably, the average roughage: concentrate ratio of the diets offered was 61:39, with decreased participation of concentrate in the diet, which resulted in no interference in the total DMI.

An increase in nutrient digestibility with the PES indicates that the diet meets the nutritional requirements of the microbial population and provides an environment with favorable conditions for microbial growth. Furthermore, nutrient digestibility is strongly related to the supply of nitrogen compounds during supplementation. These findings corroborate the results of [35], who reported an increase in the digestibility coefficients of DM, OM and CP with a PES compared with MM.

The use of additives in the PES did not compromise the digestive process. This result may be associated with the lack of influence of additives on the DMI, as changes in the DMI, for example, a decrease in the DMI, are expected to increase digestibility [36]. In some cases, the absence of differences in digestibility in cattle may be related to the nature of the diets offered, as the effect of feed intake on digestion is less pronounced with forage than with concentrate-based diets [37].

The increase in the RAN concentration with the PES may be explained by the greater intake of CP. The presence of high levels of RAN in diets with a PES suggested that the animals possibly received an adequate amount of nitrogen available in the diet [38], which was related to the greater intake of CP with supplementation. In the present study, the PES increased RAN in relation to MM (5.67 vs. 22.93 mg/dL, respectively); however, there was no effect when additives were added to the PES, which presented a mean value of 22.53 mg/dL. This concentration is above the value necessary for maximum intake of DOM (13 mg/dL) [39] and is also higher than the concentration suggested by [40] to maximize fiber intake (15 mg/dL) and similar to the value described by [41] for maximizing voluntary intake (20 mg/dL). However, when additives are used in supplements, a reduction in the concentration of RAN is desirable, as this generally leads to an increase in the efficiency of dietary protein use, which was not observed in the present study.

Considering the intake of CP (0.39 kg/day) and the RAN concentration of the animals that received only mineral supplements (5.67 mg/dL), adverse effects associated with restrictions on the degradation of insoluble fiber during the voluntary intake of forage were observed. The fact that lower intakes of DOM and total NDF digestibility were observed in steers receiving only mineral supplements corroborates these arguments.

Research has suggested that SUN concentrations greater than approximately 5 to 9 mg/dL indicate excessive nitrogen intake and nitrogen wastage [42,43]. In the diets supplemented with SUN or supplemented with additives, the SUN concentrations were above 9 mg/dL, suggesting that the CP concentration in the diets used may be above the minimum concentration necessary for the animals.

NUS levels are proportional to the concentration of RAN, and the concentration is directly linked to the level of protein and energy available in the supplementary material [44]. In this study, the higher concentration of SUN in the presence of the PES was possibly caused by the concentration of RAN. According to [45], a higher concentration of RAN causes a greater transfer of nitrogen to the bloodstream, and consequently, there is an increase in SUN. A similar response was observed for urinary nitrogen excretion, as excess ammonia results in greater nitrogen excretion via urine.

In a study of grazing beef heifers, ref. [28], there was no effect on blood parameters evaluated after supplementation with 200 mg/day monensin, which corroborates the results of the present study. The lack of effect of the PES in combination with monensin may be associated with the low nutritional value of the forage available to the animals, which could limit the increased production of propionate. However, ref. [46], when supplementing beef heifers with monensin, observed a greater concentration of SUN than when supplementing without additives. This increase may be associated with a decrease in proteolysis with increased use of nitrogen by changing the site of protein digestion [47].

Compared with the PESs with additives, the PES did not affect the rumen pH of cattle. A large part of the diet was composed of forage (61:39 roughage: concentrate ratio), which may have resulted in a longer chewing time and consequently greater salivation, both during swallowing and rumination, contributing to the buffering effects of saliva, with a great impact on ruminal pH [48].

Retained nitrogen values (average of 50.46 g/day) in diets that receive supplements or supplements + additives can be used to estimate the average daily gain in live weight (kg) of the animals [49]. Considering that approximately 750 and 250 g/kg of meat are water and protein, respectively, and that the nitrogen-to-CP ratio is 6.25 (i.e., assuming a body protein nitrogen content of 16%), these findings imply that the estimated average daily weight gain for steers receiving supplementation in the present study was approximately 1261.50 g/day. Assuming an average daily gain of 1261.50 g/day for animals with an average body weight of 355 kg, the predicted requirement for maintenance and growth [50] of the cattle in the present study was approximately 936 g/day. Therefore, the average dietary supply of CP in the supplemented diets was approximately 1095 g/day of CP, which is 14.5% greater than the predicted CP requirements. Because of the excessive amount of CP, there was greater excretion of nitrogen via the urine.

With the use of a PES, DOM intake and CP digestibility possibly influence the increase in microbial nitrogen synthesis, considering that energy and nitrogen availability are the greatest determinants of microbial protein synthesis in the rumen [51]. The greater microbial efficiency with a PES can be explained by the similar behavior between microbial nitrogen and DOM, which increased with supplementation and resulted in greater microbial efficiency.

The use of additives in the PES did not affect nitrogen use efficiency in the present study. Reference [52], when working with functional oil at doses of 810 and 2260 mg/kg DM in grazing bulls, did not observe an effect on RAN, microbial efficiency or nitrogen retention. The lack of effect according to these authors may be related to the level of supplementation of the animals.

However, ref. [38] reported an increase in microbial efficiency in beef cattle that received concentrate and were supplemented with live yeast. The authors attributed the increase in microbial efficiency to the higher concentration of RAN, which in turn would be incorporated for microbial protein synthesis. In contrast to the findings of [53] and in the present study, no effect on the RAN concentration was observed with the addition of the yeast *Saccharomyces cerevisiae*.

## 5. Conclusions

The use of the additive sodium monensin, functional oil and active yeast in a protein–energy supplement for pasture-fed beef cattle in the rearing phase did not influence nutritional parameters, not being necessary in the formulation of supplements.

## Figures and Tables

**Table 1:** Percentage and chemical composition of the protein–energy supplement.

Item	Protein–Energy Supplement	Pasture
Ingredients (%DM)		-
Soybean meals	21.84	-
Corn	76.66	-
Urea	1.5	-
Mineral mix ^1^	2	-
Chemical composition (g/kg DM)		
Dry matter	944.5	680.3
Organic matter	938.9	934.9
Crude protein	205.3	61.9
Neutral detergent insoluble crude protein	11.7	12.8
Mineral mix	46.1	65.1
Indigestible neutral detergent fiber	7.0	307.9
Neutral detergent fiber ap ^2^	115.3	649.7

^1^ 160 g/kg calcium, 60 g/kg phosphorus, 130 g/kg sodium, 12 g/kg sulfur, 8 g/kg magnesium, 100 mg/kg iron, 70 mg/kg cobalt, 700 mg/kg copper, 67 mg/kg iodine, 460 mg/kg manganese, 22 mg/kg selenium, 4.000 mg/kg zinc. ^2^ Neutral detergent fiber corrected for ash and protein.

**Table 2:** Effect of protein–energy supplementation with or without additives on the voluntary intake of beef cattle.

Item	Treatments					SEM ^3^	*p* Value			
	MM	PES	PES + MON	PES + FO	PES + YEA		I	II	III	IV
	kg/day									
Pasture dry matter Supplement dry matter	6.12 0.08	5.76 3.60	5.40 3.60	5.88 3.60	5.46 3.60	0.240 0.287	0.3139 <0.0001	0.7206 0.1448	0.6123 0.3358	0.4901 0.4335
Completely dry matter	6.20	9.36	9.00	9.44	9.06	0.342	<0.0001	0.7206	0.6123	0.4901
Organic matter	5.72	8.77	8.42	8.88	8.47	0.322	<0.0001	0.6965	0.6046	0.4813
Crude protein	0.39	1.12	1.08	1.12	1.06	0.059	<0.0001	0.2565	0.6785	0.1409
NDFap ^1^	3.64	4.25	4.01	4.33	4.05	0.148	0.0981	0.7059	0.5884	0.4641
iNDF ^2^	1.48	1.48	1.40	1.43	1.35	0.064	0.5230	0.4099	0.917	0.5238
Digestible organic matter	2.88	5.08	5.10	5.17	5.14	0.239	<0.0001	0.9772	0.918	0.9434
	%BW									
Supplement dry matter	0.02	0.89	0.90	0.90	0.91	0.073	<0.0001	0.602	0.8716	0.8474
Pasture dry matter	1.55	1.41	1.33	1.47	1.35	0.049	0.2063	0.8244	0.5097	0.4399
Completely dry matter	1.57	2.30	2.23	2.37	2.26	0.075	<0.0001	0.9087	0.4946	0.4678

MM: mineral mixture; PES: protein–energy supplement (4 kg/animal.day); PES + MON: protein–energy supplement + monensin (120 mg/animal.day); PES + FO: protein–energy supplement + functional oil (2500 mg/animal.day); PES + YEA: protein–energy supplement + active yeast (4000 mg/animal.day). Additives were added directly to the trough. ^1^ Neutral detergent fiber corrected for ash and protein; ^2^ insoluble neutral detergent fiber corrected for ash and protein; ^3^ standard error of the mean. Contrasts I: mineral mixture versus PES; II: PES without additives versus PES with additives; III: PES with MON versus PES with natural additives (FO and YEA); IV: PES with FO versus PES with YEA.

**Table 3:** Effects of protein–energy supplementation with or without additives on the apparent digestibility of beef cattle diets.

Item	Treatments					SEM ^2^	*p* Value			
	MM	PES	PES + MON	PES + FO	PES + YEA		I	II	III	IV
	g/kg DM									
Completely dry matter	469.1	552.7	580.2	557.3	581.3	1119	0.0003	0.3305	0.6135	0.3448
organic matter	499.2	579.0	603.6	581.5	603.7	1062	0.0003	0.3742	0.5933	0.3532
Crude protein	347.1	575.2	603.9	599.7	602.6	2188	<0.0001	0.246	0.9092	0.9179
iNDF ^1^	531.1	585.6	595.2	613.0	605.7	1308	0.0322	0.5293	0.6579	0.8419
	Dietary concentration (g/kg DM)									
Digestible organic matter	461	542	565	547	565	10,271	<0.0001	0.3634	0.6470	0.4344

MM: mineral mixture; PES: protein–energy supplement (4 kg/animal.day); PES + MON: protein–energy supplement + monensin (120 mg/animal.day); PES + FO: protein–energy supplement + functional oil (2500 mg/animal.day); PES + YEA: protein–energy supplement + active yeast (4000 mg/animal.day). Additives were added directly to the trough. ^1^ Insoluble in neutral detergent fiber corrected for ash and protein, ^2^ Standard error of the mean. Contrasts I: mineral mixture versus PES; II: PES without additives versus PES with additives; III: PES with MON versus PES with natural additives (FO and YEA); IV: PES with FO versus PES with YEA.

**Table 4:** Effect of protein–energy supplementation with or without additives on metabolism and nitrogen use in beef cattle.

Item	Treatments					SEM ^4^	*p* Value			
	MM	PES	PES + MON	PES + FO	PES + YEA		I	II	III	IV
RAN ^1^ mg/dL	5.67	22.93	22.37	22.34	22.50	1670	<0.0001	0.8360	0.9858	0.9579
SUN ^2^/dL	3.95	11.52	11.35	11.80	10.34	0.646	<0.0001	0.6196	0.7041	0.1079
Rumen pH	7.15	6.99	6.95	6.95	6.90	0.045	0.0138	0.4887	0.7327	0.6461
Nitrogen (g/day)										
Intake	61.76	179.17	172.46	178.83	174.14	9479	<0.0001	0.3460	0.3732	0.3698
Fecal	40.17	74.15	68.64	71.31	72.30	2662	<0.0001	0.0820	0.1215	0.6602
Urinary	26.22	53.09	54.50	55.56	53.20	2376	<0.0001	0.4966	0.9538	0.3296
Retention	−4.63	51.93	49.32	51.95	48.64	4777	<0.0001	0.5938	0.8009	0.4627
Nitrogen efficiency (g/g)										
^3^ Retained N/N intake	−0.09	0.29	0.29	0.29	0.28	0.032	<0.0001	0.8950	0.9648	0.7769
Retained N/Absorbed N	−0.27	0.49	0.48	0.48	0.48	0.067	<0.0001	0.8420	0.9795	0.9646
Microbial nitrogen (g/day)	44.85	108.22	108.09	107.02	107.61	7132	0.0001	0.9573	0.9514	0.9681
Microbial efficiency (g microbial N/kg DOM)	100.69	130.51	130.53	130.57	131.27	4979	0.0207	0.9813	0.9753	0.9614

MM: mineral mixture; PES: protein–energy supplement (4 kg/animal.day); PES + MON: protein–energy supplement + monensin (120 mg/animal.day); PES + FO: protein–energy supplement + functional oil (2500 mg/animal.day); PES + YEA: protein–energy supplement + active yeast (4000 mg/animal.day). Additives were added directly to the trough. ^1^ Rumen ammonia nitrogen, ^2^ Serum urea nitrogen, ^3^ Nitrogen, ^4^ Standard error of the mean. Contrasts I: mineral mixture versus PES; II: PES without additives versus PES with additives; III: PES with MON versus PES with natural additives (FO and YEA); IV: PES with FO versus PES with YEA.

## Data Availability

The data presented in this study are available upon request from the corresponding author.
